# Predicting Alcohol-Related Memory Problems in Older Adults: A Machine Learning Study with Multi-Domain Features

**DOI:** 10.3390/bs13050427

**Published:** 2023-05-18

**Authors:** Chella Kamarajan, Ashwini K. Pandey, David B. Chorlian, Jacquelyn L. Meyers, Sivan Kinreich, Gayathri Pandey, Stacey Subbie-Saenz de Viteri, Jian Zhang, Weipeng Kuang, Peter B. Barr, Fazil Aliev, Andrey P. Anokhin, Martin H. Plawecki, Samuel Kuperman, Laura Almasy, Alison Merikangas, Sarah J. Brislin, Lance Bauer, Victor Hesselbrock, Grace Chan, John Kramer, Dongbing Lai, Sarah Hartz, Laura J. Bierut, Vivia V. McCutcheon, Kathleen K. Bucholz, Danielle M. Dick, Marc A. Schuckit, Howard J. Edenberg, Bernice Porjesz

**Affiliations:** 1 Henri Begleiter Neurodynamics Lab, Department of Psychiatry and Behavioral Science, SUNY Downstate Health Sciences University, Brooklyn, NY 11203, USA; 2 Department of Psychiatry, Robert Wood Johnson Medical School, Rutgers University, Piscataway, NJ 08854, USA; 3 Department of Psychiatry, School of Medicine, Washington University, St. Louis, MO 63110, USA; 4 Indiana University School of Medicine, Indianapolis, IN 46202, USA; 5 Department of Psychiatry, University of Iowa, Iowa City, IA 52242, USA; 6 The Children’s Hospital of Philadelphia, University of Pennsylvania, Philadelphia, PA 19104, USA; 7 Department of Psychiatry, University of Connecticut, Farmington, CT 06030, USA; 8 Department of Psychiatry, University of California, San Diego, CA 92103, USA

**Keywords:** alcohol use disorder (AUD), EEG source functional connectivity, default mode network, alcohol-related memory problems, random forests

## Abstract

Memory problems are common among older adults with a history of alcohol use disorder (AUD). Employing a machine learning framework, the current study investigates the use of multi-domain features to classify individuals with and without alcohol-induced memory problems. A group of 94 individuals (ages 50–81 years) with alcohol-induced memory problems (the memory group) were compared with a matched control group who did not have memory problems. The random forests model identified specific features from each domain that contributed to the classification of the memory group vs. the control group (AUC = 88.29%). Specifically, individuals from the memory group manifested a predominant pattern of hyperconnectivity across the default mode network regions except for some connections involving the anterior cingulate cortex, which were predominantly hypoconnected. Other significant contributing features were: (i) polygenic risk scores for AUD, (ii) alcohol consumption and related health consequences during the past five years, such as health problems, past negative experiences, withdrawal symptoms, and the largest number of drinks in a day during the past twelve months, and (iii) elevated neuroticism and increased harm avoidance, and fewer positive “uplift” life events. At the neural systems level, hyperconnectivity across the default mode network regions, including the connections across the hippocampal hub regions, in individuals with memory problems may indicate dysregulation in neural information processing. Overall, the study outlines the importance of utilizing multidomain features, consisting of resting-state brain connectivity data collected ~18 years ago, together with personality, life experiences, polygenic risk, and alcohol consumption and related consequences, to predict the alcohol-related memory problems that arise in later life.

## 1. Introduction

Alcohol use disorder (AUD) is a chronic, relapsing disorder [1,2] with a range of neurocognitive anomalies, including memory deficits [3]. Memory impairments, among other cognitive impairments, have been widely reported to result from heavy drinking [4,5], and may interfere with social and occupational performance [6,7]. Since the etiology of AUD and related memory problems involves multiple domains, including the combination of neurocognitive, personality, behavioral, and genomic factors [8,9,10], a better understanding of these potential predictors may assist prevention and treatment strategies.

Brain oscillations representing electrical signals of neural activity, as recorded by the electroencephalogram (EEG), index specific circuit-level mechanisms during cognitive processing [11]. Oscillatory signals in different EEG frequency bands representing communications between specific brain regions underlie memory processes, including encoding, consolidation, storage, and retrieval processes [12,13]. Studies have indicated that memory processes are supported by oscillatory dynamics and communication across the hippocampus, entorhinal cortex, and other cortical regions [13,14,15]. Both human and animal studies have implicated the theta band, generated within the hippocampus and also prevalent in the cerebral cortex, as the major frequencies associated with various memory processes [16,17]. The hippocampal theta rhythm is also involved in communication with other higher frequencies (e.g., beta and gamma oscillations) through various coupling mechanisms, including neural synchrony during sensory and cognitive processing [18,19,20,21].

Recent studies have used source localization methods, such as exact low-resolution brain electromagnetic tomography (eLORETA) [22], to compute *functional connectivity,* a measure of temporal synchrony or correlation between signals of two or more spatially separated brain regions, representing functional integration between these areas [23]. These studies use *lagged connectivity* [24] to overcome volume conduction artifacts [23,25]. While the eLORETA-based functional connectivity method has been utilized to study cognitive functioning in neuropsychiatric disorders [23,26,27,28,29], very few studies have utilized these approaches to investigate AUD [30] and none have examined alcohol-induced neurocognitive outcomes, such as memory problems. Since the default mode network supports memory functions [31,32,33,34], we employed functional connectivity across the default mode network regions to examine alcohol-induced memory problems.

AUD is a multi-factorial disorder; therefore, it is important for the predictive models of alcohol-related neurocognitive outcomes, such as memory impairment, to include features from multiple domains, including polygenic risk scores (PRS) [35,36] and personality dimensions [36,37,38,39,40,41]. The identification of important variables that will reliably predict alcohol-related memory problems in older individuals may have important implications for preventive measures. Therefore, the aim of the current study is to understand and identify various features that may have predictive value in classifying individuals with memory problems. Specifically, the goal of the present study is to identify a set of multi-domain factors that can differentiate individuals with alcohol-related memory impairments from those without, using (i) resting-EEG-based functional connectivity measures of the default mode network as derived from eLORETA, (ii) PRS related to alcohol outcomes, (iii) personality and life experience measures derived from established questionnaires, and (iv) measures of alcohol consumption and associated health consequences from the recent follow-up interview. Identifying specific default mode network functional connections underlying alcohol-induced memory problems may be useful for early preventive measures and for brain-based treatment strategies such as neuromodulation therapies for addiction [42] and memory/cognitive impairment or decline [43]. Similarly, other domains, including PRS, behavioral, personality, and clinical features, may have implications for the prevention and treatment of alcohol-induced memory problems (e.g., cognitive behavior therapy, brain stimulation, cognitive remediation, etc.).

## 2. Materials and Methods

### 2.1. Sample

The sample for the present study was drawn from a recent follow-up assessment study [44,45] of participants from the Collaborative Study on the Genetics of Alcoholism (COGA) [46,47,48]. Since its inception in 1989, COGA has collected multimodal data, primarily from families that are densely affected with AUD who were identified through probands in treatment for alcohol use problems, along with a relatively smaller subset of data from community comparison families. Participants aged 50 or older who met the lifetime criteria for alcohol dependence, as assessed with the Semi-Structured Assessment for the Genetics of Alcohol (SSAGA) [49,50], were drawn from data collected at six COGA sites. Since the study participants in the COGA sample represent a high-risk sample comprising many high-density families with multiple individuals affected with AUD in higher proportions than the general population, the findings from the current study may not be readily generalizable to other populations. Nonetheless, datasets enriched for specific clinical outcomes, such as the COGA data enriched for AUD, provide an excellent opportunity for identifying markers and predictors of these outcomes of interest. However, replication studies using other data from community samples are needed to confirm these findings in the general population. Details on the screening and selection of participants for the current study are described in the Supplemental Materials (see Section S1.1 Sample Description and Figure S1 in the Supplementary Materials). During assessment, the memory and control groups were also matched for age, sex, self-reported race, genetic ancestry, and the following alcohol use patterns assessed by their last SSAGA interview conducted ~18 years prior to the recent telephone interview (see Table 1): (i) continued high-risk drinking (men with 5+ drinks/day or 15+ drinks/week and women with 4+ drinks/day or 8+ drinks/week) and meeting the criteria for DSM-5 AUD diagnosis derived from SSAGA items (N = 68/group), (ii) low-risk drinking (fewer than 5 drinks/day for men and 4 drinks/day for women) without meeting the criteria for AUD diagnosis (N = 9/group), and (iii) abstinence from drinking (N = 17/group).

### 2.2. Recent Telephone Interview

The recent follow-up telephone interview (10–20 min) was designed to collect information regarding participants’ alcohol use and current social and health status using a 31-item questionnaire [45] administered via the REDCap system [51,52]. Details about these interview items are available in Section S1.2 of the Supplementary Materials. Three items that elicited self-reported alcohol-related memory problems have been listed in Table 2. Memory impairment was coded if the participant endorsed at least two of the three items (Table 2): the first item and either the second or third item.

### 2.3. EEG Data Acquisition and Preprocessing

Details of assessments and EEG recording in COGA, which is identical at all sites, can be found in our previous reports [46,53,54]. The EEG session that was closest to the latest SSAGA interview was used for this study. Detailed descriptions of EEG data acquisition and preprocessing steps are available in Section S1.3 of the Supplementary Materials.

### 2.4. EEG Functional Connectivity Analysis Using eLORETA

EEG functional connectivity was computed using the eLORETA software [22,55], a validated tool for localizing the electrical activity in the brain. Detailed descriptions of EEG functional connectivity analysis using eLORETA are available in Section S1.4 of the Supplementary Materials.

### 2.5. Functional Connectivity across the Default Mode Network

The default mode network regions analyzed in the study are the posterior cingulate cortex (PCC), the anterior cingulate cortex, the inferior parietal cortex, the prefrontal cortex, the lateral temporal cortex, and the hippocampal formation (see Table 3 below and Figure S2 in the Supplementary Materials), in line with the functional connectivity studies of both fMRI and EEG [28,56,57] and our previous work on default mode network [58,59].

### 2.6. Assessment of Temperament, Personality, and Alcohol Experience

The temperament, personality, and life experience data included scores from seven questionnaires and their subscales; scores included for the current study are described in Section S1.6 of the Supplementary Materials. These data were collected during the previous interviews (~18 years ago) at/around the same time as the SSAGA assessment.

### 2.7. Genomic Data and Polygenic Risk Scores (PRS)

The genotyping, imputation, and quality control of COGA genomic data have been described previously [48] and in Section S1.7 of the Supplementary Materials. Table 4 lists the publicly available Genome-wide Association Studies (GWAS) for alcohol use phenotypes, derived from studies including both individuals of European ancestry (EA) and African ancestry (AA), which were used in PRS calculations in this study.

We created PRS using PRS-CSx [63,64,65,66,67], which is a recent, validated method for cross-ancestry polygenic prediction [68]. The PRS-CSx computation method is detailed elsewhere (https://github.com/getian107/PRScsx, accessed on 1 December 2021) and is also briefly described in Section S1.7 of the Supplementary Materials.

### 2.8. Feature selection of EEG Functional Connectivity Variables

In keeping with recent machine learning approaches, including our previous study [69], we used a two-stage approach consisting of feature selection followed by a predictive algorithm using selected sets of variables [70,71,72,73,74]. A detailed description of this method is available in Section S1.8 of the Supplementary Materials.

### 2.9. Random Forests Classification Model and Parameters

The random forests classification analysis was performed using the R-packages “randomForest” [75], “caret” [76], and “randomForestExplainer” [77] to classify the memory vs. control groups using multi-domain predictors. The details of these predictors, which include 29 functional connectivity, 27 personality and life experience, 12 alcohol outcomes, and 4 PRS variables, are listed in the Materials and Methods Section of the Supplementary Materials. The random forests model, as implemented in the current study, is detailed in Section S1.9 of the Supplementary Materials.

## 3. Results

### 3.1. Feature Selection of EEG Functional Connectivity Variables

The input data for the feature selection included a total of 330 EEG functional connectivity variables consisting of 66 connectivity features for each of the 5 frequency bands. The model identified a total of 29 functional connectivity variables from multiple frequency bands connecting across the 12 default mode network seeds (Refer to Table 3 in the Methods Section and Figure S2 in Supplementary Materials). These connections included Delta—12 connections, Theta—6 connections, Alpha—4 connections, Beta—5 connections, and Gamma—2 connections. The 10-fold cross-validation for the λ_1se_ threshold included all the 29 selected features, which were included in the subsequent implementation of the Random Forests classification model. The classification performance (to differentiate individuals with memory problems from those without) of the selected features as indicated by the area under the ROC curve (AUC) was 88.48%.

### 3.2. Random Forests Classification Accuracy

The overall prediction accuracy of the random forests model when classifying the memory and control group using functional connectivity, PRS, and behavioral and clinical predictors, as estimated by the AUC, was 88.29%. The 72 predictors input in the model include 29 functional connectivity, 27 personality and life experience, 12 alcohol outcomes, and 4 PRS variables (see Materials and Methods Section of the Supplementary Materials). Additional details regarding the classification accuracy are available in Section S2.2 of the Supplementary Materials.

### 3.3. Top Significant Features Contributed to the Classification

Out of the 72 input variables of the Random Forest model (see Materials and Methods section of the Supplementary Materials for details), 29 significant features that contributed to classifying the Memory group from those from the Control group were identified: 21 default mode network connections, 4 alcohol-related items, 3 personality and life experience factors, and 1 PRS (Table 5).

**Figure 1:**
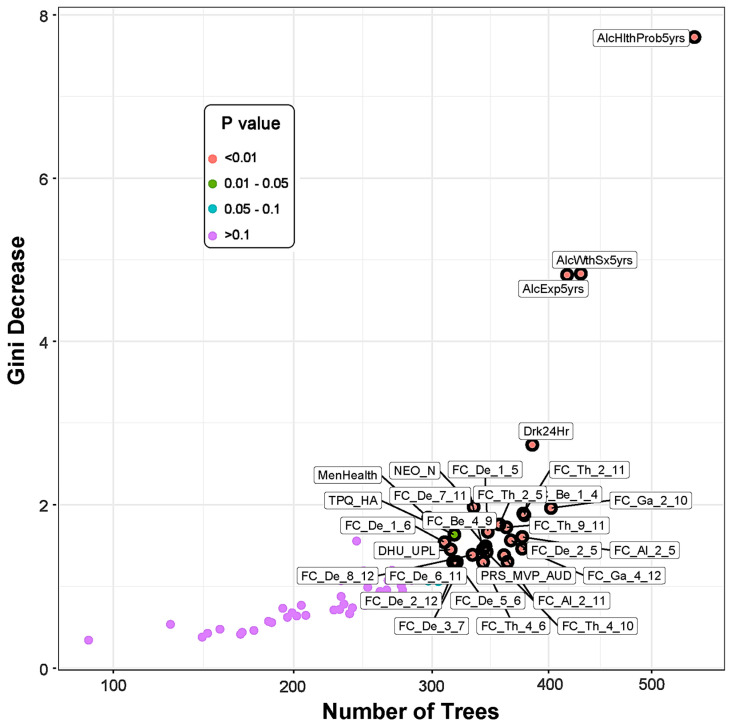
The multi-way importance plot showing the top significant variables (labeled and marked with black circles) that contributed to the differentiation of the memory group from the control subjects based on the measures Gini decrease, number of trees, and *p*-value. Features related to alcohol-related clinical/health outcomes stood top in the importance list, followed by functional connectivity, personality, and PRS measures. Note that the variables that were not significant (purple dots) are not highlighted. (See footnote of Table 5 for the list of abbreviations for the measures shown here.)

The multi-way importance plot (Figure 1) displays all of the significant variables (labeled and marked with black circles) that contributed to the classification of the *memory* group from the *control* subjects; they are ranked based on their importance for classification as derived from Gini decrease, number of trees, and *p*-value. A chart shows the distribution of minimal depth in classification against the number of decision trees (see Figure S4 in the Supplementary Materials). While both a multi-way importance plot and a distribution plot can be created for any set of random forest parameters, the importance ranking for the features is likely to be similar owing to high correlations among these parameters (see Figure S5 in the Supplementary Materials).

#### 3.3.1. EEG Source Functional Connectivity of the Default Mode Network

**Figure 2:**
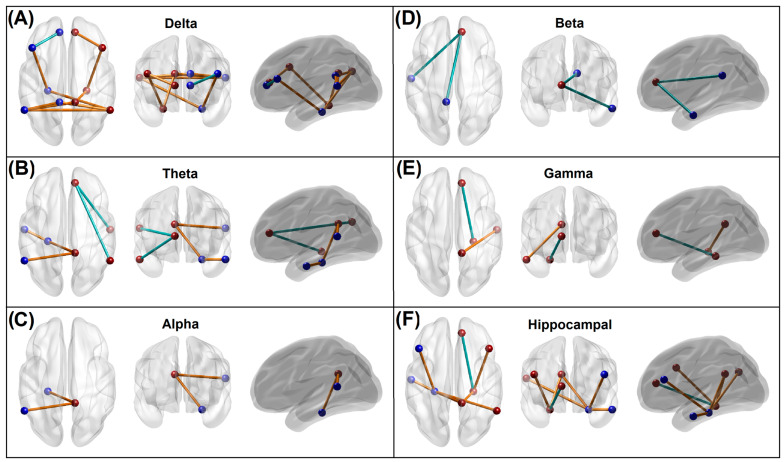
Panels (**A**–**E**): Significant default mode network connections within each frequency band, which contributed to the random forest distinction of the memory group from control individuals. The blue and brown beads represent ROIs of the left and right hemisphere, respectively, while the blue and brown lines represent hypoconnectivity and hyperconnectivity, respectively, in the memory group. Panel (**F**): Significant hippocampal connections that contributed to the memory vs. control classification. Seven of the eight hippocampal connections showed hyperconnectivity in the memory group. Note that all hypoconnected networks involved an anterior cingulate node. Refer to Figure S2 in the Supplementary Materials for the ROI locations and anatomical views/axes.

Significant default mode network connections, which contributed to the random forest distinction of the memory group from control individuals, are illustrated in Figure 2. The memory group showed a predominant pattern of hyperconnectivity across the default mode network regions, primarily constituted by the delta band (10 connections) followed by the theta band (5 connections) band, along with a lower amount of hypoconnectivity (1 in the delta band and 2 in the theta band). Other significant functional connectivity features specific to each frequency band are: (i) 9 hyperconnected paths and 1 hypoconnected path in the delta band, (ii) 3 hyperconnected and 2 hypoconnected paths in the theta band, (iii) 2 hyperconnected paths with no hypoconnected paths in the alpha band, (iv) 2 hypoconnected paths with no hyperconnected paths in the beta band, and (v) 1 hyperconnected path and 1 hypoconnected path gamma band (Figure 2, Panels A–E). The number of significant connections from each ROI node (in descending order) was as follows: R.PCC = 7; R.ACC = 6; L.PHG = 5; L.IPL = 5; R.IPL = 4; L.PCC = 3; R.PHG = 3; L.PFC = 2; R.PFC = 2; L.LTC = 2; R.LTC = 2; L.ACC = 1. The number of significant connections for the ROIs involving both hemispheres (in ascending order) was: PCC = 10; IPL = 9; PHG = 8; ACC = 7; PFC = 4; LTC = 4. Individuals from the memory group showed predominant hyperconnectivity between the hippocampal region (PHG) and other default mode network regions involving multiple frequencies, except for the beta band, compared with the *Control* group (Figure 2, Panel F). Only a single hippocampal connection (R.PHG–R.ACC) of the gamma band oscillation was hypoconnected in the memory group.

#### 3.3.2. Recent Alcohol Consumption and Health Outcomes

Significant alcohol-related health outcome variables that contributed to differentiating memory individuals from the control subjects included (i) alcohol-related health problems in the past 5 years (*Memory*_mean_ = 0.77; *Control*_mean_ = 0.01), (ii) alcohol withdrawal symptoms in the past 5 years (*Memory*_mean_ = 1.20; *Control*_mean_ = 0.11), (iii) negative experiences related to alcohol consumption in the past 5 years (*Memory*_mean_ = 2.65; *Control*_mean_ = 0.78), and (iv) the largest number of drinks within 24 h during the past 12 months (*Memory*_mean_ = 13.64; *Control*_mean_ = 6.00). Interestingly, the features concerning alcohol-related outcomes over the past 10 years, physical health outcomes, other drinking patterns, and demographic variables were not significant.

#### 3.3.3. Measures of Personality, Behavior, and Life Experiences

Out of 27 variables of personality and behavioral features, only the following 3 variables significantly contributed to the memory vs. control classification: (i) harm avoidance representing internalizing traits and negative mood states as assessed by TPQ (*Memory*_mean_ = 16.16; *Control*_mean_ = 12.61), (ii) uplift experience, indicating “feel good” aspects as assessed by DHU (*Memory*_mean_ = 51.25; *Control*_mean_ = 58.99), and (iii) neuroticism, represented by dysregulated emotions and maladjusted behaviors as assessed by NEO (*Memory*_mean_ = 59.00; *Control*_mean_ = 52.11), where higher scores mean more neurotic traits.

#### 3.3.4. Polygenic Risk Scores

PRS for the AUD diagnosis (based on the ICD codes) created using GWAS data from the MVP [60] was a significant contributor to the classification of the memory vs. control groups (*Memory*_mean_ = 8.25 × 10^−7^ and *Control*_mean_ = 7.87 × 10^−7^). PRSs for the other phenotypes, i.e., AUDIT-C scores from the GWAS of the MVP dataset [60], maximum habitual alcohol intake from the GWAS of the MVP dataset [61], and a DSM-IV alcohol dependence diagnosis from the GWAS of the PGC dataset [62], were not significant contributors in the classification.

### 3.4. Correlations across Significant Predictors

**Figure 3:**
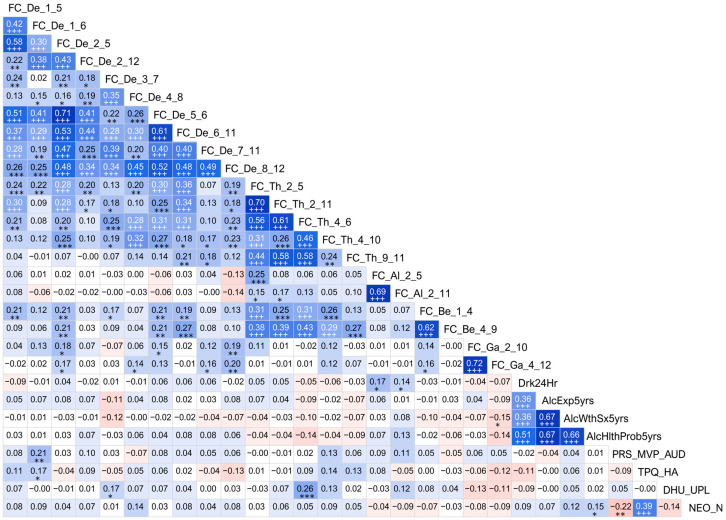
Correlation matrix showing associations among the top significant variables. Values of the cells in red/pink shades represent negative r-values, and those in blue/cyan shades indicate positive r-values between variables that correspond to the vertical and horizontal axes. A darker color represents a higher magnitude of r-values. Significant correlations (before Bonferroni correction) are marked with asterisks in black font (* *p* < 0.05; ** *p* < 0.01; and *** *p* < 0.001), and those that survived Bonferroni correction are marked with a triple plus sign (+++) in white font. For the abbreviations in the variable labels, see the footnote of Table 5.

An exploratory (descriptive) analysis of correlations among the top significant variables is shown in Figure 3. As shown in the correction matrix, there were significant positive correlations among the functional connectivity variables within and between different frequency bands. Overall, most of the low-frequency connections in the delta and theta frequencies were highly correlated with one another. Specifically, those connections that shared a common node showed much higher correlations with each other than with other connections, regardless of their frequency bands. Beta band connections had significant positive correlations between themselves as well as with low-frequency connections, especially theta band connections. However, alpha and gamma band connections showed significant correlations only within the frequency and not across the frequencies. Highly significant positive correlations were observed among the alcohol-related health consequences. Among the personality factors, there was a significant positive correlation between neuroticism and harm avoidance. However, no significant correlations were observed across the domains (e.g., functional connectivity vs. personality, or functional connectivity vs. alcohol-related features).

## 4. Discussion

The current study suggests that alcohol-related memory problems can be predicted using a multi-domain set of features from neural, behavioral, genomic, and alcohol-related measures in a machine-learning framework. It was found that the memory group showed a predominant pattern of hyperconnectivity across the default mode network regions, including the hippocampal subnetworks, while showing hypoconnected anterior cingulate cortex subnetworks; these results were based on the EEG recorded about 18 years ago. Features from other domains that significantly contributed to the classification were (i) higher counts of alcohol-related consequences during the past five years, such as health problems, other alcohol-related adverse past negative experiences, withdrawal symptoms, and a higher max number of drinks (the largest number of drinks per day), (iii) personality factors such as high neuroticism, high harm avoidance, and low rates of positive/uplifting experiences, and (iv) high genetic liability, as reflected in variations in PRS for AUD across the memory and control groups. It should also be noted that the classification accuracy was better for the control individuals (85/94 = 90.43%) than for the memory group (68/94 = 72.34%). Although there are many possible reasons for this, we speculate that the memory group may have high variability in their clinical presentations and/or neurocognitive functioning.

### 4.1. Altered Functional Connectivity in the Memory Group

The findings for resting-state EEG connectivity showed that the subjects with alcohol-related memory problems, relative to the matched controls, showed (i) a predominant pattern of hyperconnectivity of low-frequency (delta and theta) oscillations across most of the default mode network cortical regions, (ii) hyperconnected hippocampal sub-networks in multiple frequency bands, and (iii) hypoconnectivity in subnetworks involving anterior cingulate cortex hub regions. In general, alterations in brain networks (in both low and high frequencies) due to alcohol-induced memory deficits could be interpreted as compromised memory engrams and changes in neural plasticity during the encoding and recall processes. The neural basis of memory processes was first theorized by Richard Semon’s engram theory [78] and Donald Hebb’s *synaptic plasticity theory* [79], and the body of literature on memory functions is vast and spans several decades. The connectivity differences observed between the memory and control groups are discussed below in light of findings from the literature, as well as from our previous studies.

#### 4.1.1. Predominant Hyperconnectivity of Low-Frequency Oscillations in the Memory Group

The finding that individuals with alcohol-induced memory problems during their recent interview (i.e., the memory group) manifested a predominant pattern of hyperconnectivity across the default mode network nodes in their resting state EEG (Figure 2) may indicate aberrations in neural communication. Specifically, EEG hyperconnectivity may indicate a brain signature related to an early stage of cognitive decline, possibly leading to dementia [80]. While it is far from clear whether the EEG-based functional connectivity findings are attributable to a specific diagnosis or outcome, increased EEG connectivity during the resting state may be a sign of abnormal brain communication, since studies have reported this feature in several neuropsychiatric disorders. For example, individuals with schizophrenia had increased EEG coherence in delta and theta bands relative to controls [81]. Similarly, patients with major depressive disorder exhibited significantly higher EEG coherence in several frequencies, including delta and theta bands, as compared to controls [82]. Such alterations in resting-state EEG connectivity in slow rhythms (delta and theta) have also been reported in childhood developmental disorders, such as autism spectrum disorders [83] and specific learning disorders [84]. On the contrary, healthy aging is marked by decreased slow frequency activity (band power) in the delta and theta bands during the resting state [85], as well as by reduced EEG network connectivity [86]. On the other hand, while performing tasks, both delta and theta band oscillations predominantly contribute to the generation of P300 or P3 [87], a prominent event-related potential (ERP) component that is a marker of contextual neural processing, the amplitude of which is reduced abnormally in individuals with and/or at risk for AUD, who have shown reduced amplitudes [9]. Interestingly, slow delta and theta oscillations are often found to be attenuated during task performance in individuals with chronic AUD relative to healthy individuals [88], while these slow theta oscillations are also involved in episodic memory maintenance processes during cognitive processing [89].

At the neural level, it is possible that the hyperconnectivity seen in the memory group may contribute to aberrant synaptic pruning in specific cortical regions [90] in these individuals, who also report having increased alcohol-related consequences compared to the comparison group. It is also possible that damage to a specific network can enhance connectivity across other regions that are anticorrelated to the damaged network, such as occurs in neurodegenerative conditions [91]. Physiologically, alcohol can impact pre- and postsynaptic mechanisms during the secretion/recycling of neurotransmitters, leading to the disruption of excitatory and inhibitory neurotransmission [92,93], potentially caused by the detrimental effects of alcohol on glial cells [94]. Recent animal studies confirm that chronic and heavy alcohol consumption can cause aberrant synaptic pruning and the substantial loss of excitatory synapses in the prefrontal cortex, resulting in disruption of brain connectivity and dysregulated neural communication across the cortical networks [95]. However, it remains to be confirmed whether the connectivity differences observed in the memory group are the direct consequence of alcohol consumption or indicators of predisposed genetic risk in these individuals or the interaction of both.

#### 4.1.2. Hyperconnectivity across the Hippocampal–Cortical Networks in the Memory Group

Our findings reveal that individuals who experience alcohol-related memory problems also show a predominant pattern of hyperconnectivity across the hippocampal network in their resting EEG, which was recorded about 18 years ago. Specifically, these hyperconnected hippocampal networks (seven out of eight connections) involved the bilateral PHG, bilateral PFC, left LTC, right PCC, and right IPL nodes, and spanning delta, theta, and alpha bands (Figure 2, Panel F). Furthermore, the majority of the hyper-connected paths (six out of seven connections) represented low-frequency (delta/theta) oscillations. Although direct evidence linking the EEG-based hyperconnectivity of the parahippocampal–cortical network to alcohol-related memory problems is lacking in the literature, some of the available findings may help interpret the results of the present study. Interestingly, intracranial EEG recordings taken at the hippocampus and medial temporal regions revealed the existence of independent delta/theta rhythms in different subregions of the human hippocampus and surrounding cortical regions that are associated with memory encoding and retrieval [96]. Therefore, it is possible that dysregulation (i.e., hyperconnected low-frequency paths) in the hippocampal–cortical network, which underlies memory processing [97], may have directly contributed to the alcohol-related memory problems in the memory group. At the neural level, elevated hippocampal resting-state connectivity may be associated with age-related decline in the white matter integrity of the fornix, as well as deficient neurocognitive function, in human adults [98]. Converging findings indicate that memories of recent events underlie the dynamic interplay across multiple cortical brain regions and networks, in which the hippocampus acts as a hub, integrating information from these subnetworks [99]. Recent studies reveal hippocampal involvement in the default mode network activity. The default mode network may mediate interactions between the hippocampus and the neocortex in memory formation and replay [100]. A large neuroimaging study revealed that subregions within the default mode network contain fornix fibers from the hippocampus, thus relating the network to its memory functions [101]. Specifically, a hyperconnected bilateral hippocampal-prefrontal network of slow frequency (delta band) may indicate a dysregulated long-range neural communication involving learning and memory processes, as these networks are crucial for the coordination of activity during memory-guided decision-making [102]. Further, the theta band hyperconnectivity of the left hippocampal with the left temporal cortex and right PCC in the memory group may indicate disturbances in verbal [103] and episodic memory [104], respectively. This finding regarding theta band hippocampal connectivity is important, as the hippocampal theta rhythm is critical for the optimal functionality of memory networks [105]. It may also be interesting to note that theta band hyperconnectivity across cortical regions was also observed in the APOE-4 carriers of patients with Alzheimer’s disease [106]. Lastly, it should be noted that a single connection with decreased connectivity at the gamma band in the memory group was observed between ACC and PHG in the right hemisphere. Weaker resting-state connectivity between the hippocampus and ACC may suggest the disruption of mood regulation [107], possibly due to compromised structural connectivity between these major structures [108]. Another explanation for the lower connectivity between the hippocampus and ACC in the memory group [109] is the presence of alcohol-induced microstructural alterations in neuronal fiber tracts connecting brain structures in AUD individuals [110], as occurs in patients with traumatic axonal injury, causing damage to axonal fiber tracts across and within the hemispheres, including the hippocampal-cortical bundles [111]. As mentioned earlier, given that the memory group reported more instances of heavy drinking and alcohol-related health consequences than the control group, it is expected that neuronal damage, including the compromised hippocampal–cortical connectivity, is more pronounced in these individuals, resulting in memory problems along with other neurocognitive and health issues. In sum, it is possible that alcohol-induced hippocampal atrophy [112] may underlie the disruption of the cortical–hippocampal network that underpins memory formation and retrieval processes [113,114].

#### 4.1.3. Hypoconnectivity across the Anterior Cingulate Hub Networks in the Memory Group

The findings of the present study also reveal that, in addition to the predominant hyperconnectivity across the default mode network nodes in multiple frequencies, the memory group exhibited six hypoconnected paths (i.e., with reduced connectivity strength) across the bilateral ACC and other cortical regions (left PFC, bilateral LTC, R.IPL, left PCC, and right PHG) in all frequency bands except the alpha band. All connections, except those in the beta band, were intra-hemispheric. Broadly, since the ACC hub networks within the default mode network are associated with the prediction of outcomes for a given choice [115], the planning of future actions [116], and social cognition [117], the hypoconnectivity of the ACC with other cortical regions, including the hippocampal region, may indicate disrupted neural communication leading to less efficient action plans and decision making. The ACC also contributes to reward-based action selection or decision making [118,119,120], as well as the monitoring of action, conflict, error, and outcomes [121,122,123,124]. In our previous study on EEG source connectivity in abstinent AUD individuals [58], we also reported hypoconnected prefrontal nodes (PFC and ACC) relaying other cortical regions (LTC, IPL, and PHG) suggesting weaker top-down processing.

Specifically, the hypoconnected ACC–PFC subnetwork in the memory group may suggest compromised top-down cognitive control mediated by the PFC, such as that observed in individuals who are addicted to drugs [125]. On the other hand, reduced connectivity of the ACC with the LTC in the memory group may represent impaired semantic memory processing related to personally relevant action plans in these individuals, as the LTC is related to short-term verbal memory and language processes [126,127] as well as conceptual representations of actions and behaviors [128,129]. Furthermore, hypoconnectivity between the ACC and the IPL in the right hemisphere may indicate a lack of spatial and computational processing for the task at hand, as dictated by the role of the right IPL in spatial attention and mathematical cognition [130]. Taken together, these alterations in the brain network may underpin alcohol-induced memory deficits in individuals from the memory group, who also exhibit more health problems due to their chronic and/or hazardous alcohol consumption (see Section 4.2 below).

### 4.2. Alcohol Consumption and Health Problems in the Memory Group

The foremost predictors of memory problems as revealed by the random forests model were alcohol-related consequences during the past five years, such as health problems, past negative experiences, and withdrawal symptoms, and the largest number of drinks per day. This finding indicates that the individuals with alcohol-related memory problems not only consumed larger quantities of alcohol during the last five years, but also suffered drinking-related adverse consequences, such as withdrawal symptoms, negative experiences, and health issues. It is quite possible that the memory problems experienced by the individuals from the memory group could be one of the health and neurocognitive outcomes of chronic and/or hazardous alcohol consumption as supported by the relevant literature [131,132,133]. Relatedly, a great deal of research documents alcohol-induced brain damage and cognitive impairments, including memory deficits, in chronic and hazardous drinkers [134,135,136]. Taken together, alcohol-induced memory problems could be a part of a larger picture of severe brain damage in chronic and/or heavy users of alcohol. Future longitudinal studies combining both structural and functional MRI, along with various EEG and neuropsychological measures, may clarify the exact nature of alcohol-induced neurocognitive deficits.

### 4.3. Personality Features in the Memory Group

Among the host of personality and life experience factors included in the random forests model, only three factors, namely, harm avoidance, neuroticism, and uplift experiences, were identified as key features that contributed to differentiating the memory group from the controls. Our findings suggest increased harm avoidance in the memory group, evidenced by more evidence of internalizing traits and negative mood states in these individuals. Although past studies have shown mixed findings for the harm avoidance subscale of the TPQ in predicting AUD/SUD and risk [38,137], some recent studies have associated these internalizing traits with the harmful use of alcohol and other substances [138,139] and with the risk of developing AUD [140,141,142]. Interestingly, alcohol and other psychoactive substances are often used to self-medicate negative mood states such as depression [143,144]. Furthermore, higher neuroticism in the memory group may be related to a variety of alcohol-related outcomes, including relapse [145]. Additionally, neuroticism has been associated with the ineffective use of coping strategies [146], while also mediating the relationship between AUD and neural connectivity [147]. Empirically, neuroticism has also been found to be associated with internalizing factors related to the lifetime diagnosis of mood and anxiety [148]. On the other hand, individuals from the memory group also reported fewer uplifting experiences than the comparative controls, reflecting less pleasurable experiences at work and home. A lack of adequate uplifting experiences represents a lower buffer against stress and reduced coping abilities [149], which can also contribute to both AUD [146,150] and internalizing outcomes such as depression [151,152]. Alternatively, negative mood states may lead to the assessment of fewer experiences as uplifting. Taken together, it is clear that personality- and life-experience-related factors are important determinants in alcohol-related outcomes, possibly mediated by neural and stress–coping dyad mechanisms. However, further studies are needed to elucidate the specific mechanisms involved in the complex etiological pathways of risk, symptoms, and recovery in AUD and related disorders.

### 4.4. Genomic Risk in the Memory Group

The only significant PRS measure in the random forest model for classifying the memory and control groups was derived from the MVP study of DSM-5 AUD, suggesting the importance of AUD-PRS, rather than consumption-related PRS, in predicting neurocognitive outcomes such as alcohol-induced memory problems. This may partly be because individuals from both the memory and control groups had a lifetime diagnosis of DSM-IV alcohol dependence. While the DSM-IV alcohol dependence PRS derived from the PGC was not found to be significant, it is possible that this could be because of its relatively smaller GWAS sample size, compared to that of the MVP dataset, as well as the presence of fewer participants of non-European ancestry in the discovery GWAS (see Table 4) and/or the more inclusive diagnosis of DSM-5 AUD compared to DSM-IV AD. Nevertheless, the finding that AUD-PRS significantly contributed to the classification suggests that alcohol-induced memory issues, at least in part, are associated with genomic liability. In general, family studies, twin studies, and GWAS have all demonstrated the heritability of AUD [153,154,155] and the utility of PRS to identify and quantify the risk of developing AUD and related outcomes [65,67,156]. Recently, Lai et al. [67] reported that individuals with AUD had higher PRS than controls and the PRS magnitude increased as the number of DSM-5 diagnostic criteria increased. Furthermore, PRS for alcohol dependence was found to be associated with neural connectivity [36,157] and cognitive functions, such as verbal fluency, vocabulary, digit-symbol coding, and logical memory [158], as well as brain structure [159]. Unfortunately, PRS factors related to neurocognitive phenotypes, which could have improved the predictive model, were not included in the study due to a lack of neurocognitive GWAS on AA populations when calculating PRS-CSx for the study sample. Further studies using neurocognitive PRS in multi-ethnic samples are needed to ascertain and quantify the genomic contribution of alcohol-induced memory problems for predictive purposes.

### 4.5. Correlations among the Significant Features

It may be of interest to understand how the significant features, which contributed to the differentiation of memory individuals from controls, are related to each other. As shown in Figure 3, the correlation matrix revealed some interesting associations. Most obviously, most of the low-frequency connections in the delta and theta frequencies were highly correlated with one another. As mentioned earlier (Section 4.1.2), hippocampal EEG oscillations are mainly represented by delta and theta frequencies, which interact with each other in the memory processes, such as in mnemonic encoding and retrieval [96]. Empirically, it is known that delta and theta rhythms are not only correlated with each other but are involved in hippocampal–prefrontal communication, which underlies memory and other higher-order cognitive functions such as executive functions [160,161]. Another interesting finding was that the connections that shared a common node (brain region) were also significantly correlated with each other, regardless of their frequency band. It is possible that the common node forms a subnetwork that can facilitate information flow across the regions of the subnetwork as well as other connected regions in the brain [162]. Further, correlational results also showed that the beta band connections had highly significant correlations with other connections within the same frequency as well as among low-frequency connections (*p* < 0.001), especially with the theta band connections (*p* < 0.001 and survived Bonferroni correction). This could be because low frequencies (delta/theta) synchronously work together with high frequencies (beta/gamma) during cognitive processing, including working memory processes [163,164,165]. However, alpha and gamma band connections showed only within-frequency correlations but no cross-frequency correlations, partly because the magnitude of correlations is smaller and requires more statistical power to identify meaningful alpha–gamma associations.

Correlations among the alcohol-related outcome variables were also found to be highly significantly related with one another, which is in line with the research showing heavy and high-intensity drinking is associated with alcohol-related negative consequences such as withdrawal symptoms and health issues [166,167]. Furthermore, the significant positive correlation between the two personality traits, namely, neuroticism and harm avoidance, is also backed by the evidence that both traits underlie negative emotions such as fear, shyness, and worry and are regulated by serotonin and opiate pathways [168]. Lastly, it was a rather unexpected finding that there were no highly significant correlations across the domains (e.g., functional connectivity vs. personality), likely because of very low levels of correlation across the domains due to insufficient statistical power to detect the subtle associations among features from different categories of predictors.

### 4.6. Limitations and Suggestions

While this is the first multi-modal study that uses EEG-based source connectivity to examine alcohol-related memory problems, which is an important alcohol-related neurocognitive outcome, it has some limitations: (i) the sample size of the study groups is rather small and the findings are therefore only preliminary, (ii) while the groups are matched based on important variables, stratified analyses based on age, sex, and self-reported race and genetic ancestry, may identify more relevant features specific to each category; (iii) some of the variables were not considered for matching (e.g., memory status during baseline, relatedness among group members, comorbid diagnoses such as substance use, anti-social personality disorder, attention-deficit hyperactivity disorder, etc.), which may have impacted the results; (iv) the memory problems reported by the study sample can be heterogeneous and the assessment of alcohol-related memory problems was only based on oral self-reports and not a psychometric measure; studies that are currently underway are assessing this sample with comprehensive neurocognitive assessments, including memory function, and will be more objective and quantitative; (v) the study does not consider genomic or other trait-related baseline effects that could have influenced the results, and future large-scale studies may consider this aspect in the study design; (vi) for the current sample, recent EEG recordings and neurocognitive assessments including memory function that are missing in the current study, but are underway in our lab, will further add to our predictive modeling; (vii) other specific networks and regions related to memory (e.g., attention and memory networks) are not explored in the current study, although studies are underway in our lab to explore these networks; (viii) PRS for neurocognitive phenotypes, including memory functions, were not included due to lack of availability of multi-ethnic GWAS data. Future studies may attempt to overcome the shortcomings of the study by using larger sample size and stratified analyses, longitudinal designs, multimodal imaging (e.g., fMRI, DTI), and neurocognitive PRS data.

## 5. Conclusions

Our study has elucidated the key multimodal features of brain connectivity, personality, life experiences, and genomic and alcohol-related measures that can serve as predictors of later alcohol-related memory problems, which occur after about 18 years. Dysregulated brain connectivity, computed from the EEG data collected 18 years ago, in the form of hyper- and hypo-connectivity in specific subnetworks and including the hippocampal–cortical connections, represents a potential neural correlate of alcohol-related memory problems. Personality and life experience features, such as higher neuroticism and excessive harm avoidance, as well as the presence of fewer uplifting experiences in daily life, also contributed to distinguishing individuals with memory problems from the controls. Importantly, alcohol-related negative consequences during the past 5 years, such as health problems, past negative experiences, withdrawal symptoms, and the largest number of drinks in a day during the past 12 months, were the top predictors of memory problems. These findings will require confirmation in future studies to: (i) validate these multi-domain features for use in the early identification of individuals who may develop alcohol-induced memory problems, especially chronic and/or heavy drinkers; and (ii) use EEG-source connectivity measures to further identify/validate the specific targets of brain networks underlying AUD-related outcomes in general and memory deficits in particular, in order to propose neuromodulation-based treatments (e.g., transcranial magnetic stimulation) as guided by the neural signatures related to dysregulated brain networks in the affected individuals. However, the study has many limitations, and the results are only preliminary, warranting large-scale future studies that can confirm these findings by adopting better experimental designs using predictive modeling.

## Figures and Tables

**Table 1:** Demographic characteristics, AUD remission status during the latest SSAGA interview before the follow-up telephone interview, and details of alcohol consumption from the recent telephone interview for the EEG functional connectivity analysis.

Variable	Measure/Category	Parameter	Study Group	
			Memory (N = 94)	Control (N = 94)
**Age during assessment**	EEG *	Min–Max	29.21–60.71	28.17–62.19
		Mean (SD)	39.42 (6.18)	40.11 (6.74)
	Follow-up interview	Min–Max	50.55–81.86	50.34–81.49
		Mean (SD)	57.84 (5.77)	58.75 (6.07)
**Sex**	Male	N (%)	52 (55.30)	52 (55.30)
	Female	N (%)	42 (44.70)	42 (44.70)
**Self-reported race**	White	N (%)	67 (71.30)	67 (71.30)
	Black	N (%)	24 (25.50)	24 (25.5)
	Other	N (%)	3 (3.20)	3 (3.20)
**Genetic ancestry**	European	N (%)	63 (50.40)	62 (49.60)
	African	N (%)	23 (47.92)	25 (52.08)
	Other	N (%)	8 (53.33)	7 (46.67)
Alcohol use pattern during the latest SSAGA interview *	AUD diagnosis	N (%)	68 (72.30)	68 (72.30)
	Low-risk drinking	N (%)	9 (9.60)	9 (9.60)
	Abstinence	N (%)	17 (18.10)	17 (18.10)
Time lag **	Years	Mean (SD)	18.42 (3.84)	18.63 (3.90)

* The latest SSAGA interviews were also closer in time to the EEG recording used for the current study. Note that the SSAGA interview is longer and more comprehensive than the recent follow-up phone interview. ** Time lag (years) between the latest past (baseline) assessments (EEG, SSAGA, and clinical/personality) and the recent follow-up telephone interview.

**Table 2:** Items related to memory problems in the follow-up interview questionnaire.

Domain	Question	Memory-Related Response *
**Alcohol-related memory problems**	Compared to most people your age, is your memory currently better, about the same, or worse than theirs?	Worse
	** There are several other health problems that can result from heavy drinking. In the last 5 years did drinking: (check all that apply)	Impair your memory even when you were not drinking (not including blackouts)?
	** There are several other health problems that can result from heavy drinking. In the last 10 years did drinking: (check all that apply)	Impair your memory even when you were not drinking (not including blackouts)?

* Response option related to memory problems. ** These items are the same for the categories eliciting alcohol use during the past 5 years and the past 10 years.

**Table 3:** Regions of interest (ROIs), region code/abbreviation, Brodmann area (BA), and the MNI coordinates for the default mode network are listed.

ROI	Region Name	Region Code	BA	MNI (X)	MNI (Y)	MNI (Z)
**1**	Left posterior cingulate cortex	L.PCC	23	−10	−45	25
**2**	Right posterior cingulate cortex	R.PCC	23	10	−45	25
**3**	Left anterior cingulate cortex	L.ACC	32	−10	45	10
**4**	Right anterior cingulate cortex	R.ACC	32	10	45	10
**5**	Left inferior parietal lobule	L.IPL	40	−55	−55	20
**6**	Right inferior parietal lobule	R.IPL	40	55	−55	20
**7**	Left prefrontal cortex	L.PFC	46	−45	25	25
**8**	Right prefrontal cortex	R.PFC	46	45	25	25
**9**	Left lateral temporal cortex	L.LTC	21	−55	−15	−20
**10**	Right lateral temporal cortex	R.LTC	21	55	−15	−20
**11**	Left parahippocampal gyrus	L.PHG	36	−25	−30	−20
**12**	Right parahippocampal gyrus	R.PHG	36	25	−30	−20

**Table 4:** List of polygenic risk scores (PRS) datasets from recently published GWAS.

Phenotype	Discovery Sample/Consortium	Sample Size	
		EA	AA
AUD diagnosis (ICD-9/ICD-10)	MVP [60]	202,004	56,648
AUDIT-C symptoms	MVP [60]	200,680	56,495
Max alcohol intake	MVP [61]	126,936	17,029
Alcohol dependence (DSM-IV)	PGC [62]	46,568	6280

**Table 5:** Random forest importance parameters and direction of significance for the top significant variables (*p* < 0.05) are shown. The variables are sorted based on the Gini decrease. Details of these features are available in the Materials and Methods Section of the Supplementary Materials.

Feature	Measure/Source	Gini Decrease	Accuracy Decrease	# Trees	# Nodes	Times a Root	Min. Depth	*p* Value	Direction
**AlcHlthProb5yrs**	FU Interview	7.7281	0.0449	545	610	111	2.3303	8.26 × 10^−47^	MEM > CTL
**AlcWthSx5yrs**	FU Interview	4.8291	0.0196	430	459	109	3.8230	4.09 × 10^−13^	MEM > CTL
**AlcExp5yrs**	FU Interview	4.8134	0.0176	417	468	95	4.0144	1.42 × 10^−14^	MEM > CTL
**Drk24Hr**	FU Interview	2.7318	0.0097	385	440	70	5.0280	2.75 × 10^−10^	MEM > CTL
*NEO_N	Questionnaire	1.9701	0.0029	334	382	47	5.6475	6.84 × 10^−4^	MEM > CTL
**FC_Ga_2_10**	R.PCC–R.LTC	1.9574	0.0019	402	486	5	5.5047	1.02 × 10^−17^	MEM > CTL
**FC_Th_2_11**	R.PCC–L.PHG	1.8902	0.0020	377	463	11	5.7415	9.38 × 10^−14^	MEM > CTL
**FC_Be_1_4**	L.PCC–R.ACC	1.8699	0.0030	378	463	6	5.8232	9.38 × 10^−14^	CTL > MEM
**FC_Th_2_5**	R.PCC–L.IPL	1.7564	0.0039	356	424	16	5.8446	3.53 × 10^−8^	MEM > CTL
**FC_Th_9_11**	L.LTC–L.PHG	1.7206	0.0010	362	437	17	5.8282	7.15 × 10-10	MEM > CTL
**FC_De_1_5**	L.PCC–L.IPL	1.6655	0.0011	346	412	12	6.0057	9.11 × 10^−7^	MEM > CTL
*TPQ_HA	Questionnaire	1.6312	0.0026	318	363	37	6.1333	1.44 × 10-02	MEM > CTL
**FC_Al_2_5**	R.PCC–L.IPL	1.6034	0.0013	376	455	9	5.9314	1.72 × 10^−12^	MEM > CTL
**FC_De_2_5**	R.PCC–L.IPL	1.5614	0.0004	366	437	18	5.8339	7.15 × 10^−10^	MEM > CTL
**FC_De_1_6**	L.PCC–R.IPL	1.5384	0.0009	310	383	27	6.2101	5.68 × 10^−4^	MEM > CTL
**FC_Be_4_9**	R.ACC–L.LTC	1.4901	0.0009	344	402	12	6.2038	1.05 × 10^−5^	CTL > MEM
**FC_Ga_4_12**	R.ACC–R.PHG	1.4605	0.0016	376	451	3	5.6709	6.99 × 10^−12^	CTL > MEM
**FC_De_7_11**	L.PFC–L.PHG	1.4543	0.0019	342	407	13	6.1891	3.19 × 10^−6^	MEM > CTL
*DHU_UPL	Questionnaire	1.4497	0.0021	315	368	15	6.4736	7.06 × 10^−3^	CTL > MEM
**FC_Th_4_10**	R.ACC–R.LTC	1.4211	0.0006	345	422	8	6.2084	6.21 × 10^−8^	CTL > MEM
**FC_De_8_12**	R.PFC–R.PHG	1.3844	0.0010	333	394	15	6.0851	6.29 × 10^−5^	MEM > CTL
**FC_Al_2_11**	R.PCC–L.PHG	1.3805	0.0006	360	443	3	6.2337	1.04 × 10^−10^	MEM > CTL
**PRS_MVP_AUD**	PRS	1.2987	0.0002	363	432	1	6.2696	3.35 × 10^−9^	CTL > MEM
**FC_De_5_6**	L.IPL–R.IPL	1.2964	0.0009	320	378	11	6.4012	1.40 × 10^−3^	MEM > CTL
**FC_De_6_11**	R.IPL–L.PHG	1.2959	−0.0001	317	381	10	6.3433	8.21 × 10^−4^	MEM > CTL
**FC_Th_4_6**	R.ACC–R.IPL	1.2955	0.0002	342	404	2	6.3120	6.59 × 10^−6^	CTL > MEM
**FC_De_2_12**	R.PCC–R.PHG	1.2581	0.0007	319	380	9	6.4407	9.83 × 10^−4^	MEM > CTL
**FC_De_4_8**	R.ACC–R.PFC	1.1741	0.0015	315	364	6	6.5837	1.26 × 10^−2^	MEM > CTL
**FC_De_3_7**	L.ACC–L.PFC	1.1278	0.0000	319	391	6	6.7618	1.18 × 10^−4^	CTL > MEM

Abbreviations: FC—functional connectivity; De—Delta; Th—Theta; Al—Alpha; Be—Beta; Ga—Gamma. Numbers in functional connectivity variables: 1–12 of the default mode network; AlcHlthProb5yrs—alcohol-related health problems in the past 5 years; AlcWthSx5yrs—alcohol withdrawal symptoms in the past 5 years; AlcExp5y—alcohol related negative experiences (symptoms) related to alcohol consumption in the past 5 years; Drk24Hr—the largest number of drinks in 24 h during the past 12 months; PRS_MVP_AUD—PRS derived from the MVP GWAS of AUD; * These measures were derived from the following personality and life experience questionnaires: TPQ_HA—harm avoidance assessed by TPQ questionnaire; DHU_UPL—uplift assessed by DHU questionnaire; and NEO_N—neuroticism assessed by NEO questionnaire. See Table 3 in Methods and Figure S2 in the Supplementary Materials for the details of the ROIs of the default mode network. MEM—memory group; CTL—control group.

## Data Availability

The COGA data used in the current study are available from the website <https://zork5.wustl.edu/coganew/contacts.html, accessed on 1 May 2020> upon written request. Details regarding access to COGA data are available through the National Institute of Alcoholism and Abuse at http://www.niaaa.nih.gov/research/major-initiatives/collaborative-studies-geneticsalcoholism-coga-study#Acess, accessed on 1 May 2020. COGA data are also available from the publicly accessible dbGAP database at http://www.ncbi.nlm.nih.gov/gap/?term=COGA, accessed on 1 May 2020 [IDs: phs000092.v1.p1, phs000125.v1.p1, and phs000763.v1.p1].

## Supplementary Materials

The following supporting information can be downloaded at: https://www.mdpi.com/article/10.3390/bs13050427/s1, Figure S1: Flowchart showing the selection process for the study sample; Figure S2: The default mode network nodes included for the computation of functional connectivity; Figure S3: ROC curve (red line) derived from the Random Forests model to classify *Memory* and *Control* individuals using functional connectivity, PRS, and clinical and behavioral predictors; Figure S4: The distribution of minimal depth among the trees of the forests for each feature is color-coded for different levels of minimal depth; Figure S5: Concordance of rankings between any two Random Forests parameters; Table S1: List of variables from the follow-up interview schedule (N = 12) included in the random forest classification model; Table S2: List of variables (N = 27) for the personality and life experiences questionnaires; Table S3: The list of functional connectivity variables (N = 29) that were identified by the feature selection method and included in the random forest classification analysis; Box S1: Concepts and parameters used in the random forest classification method. References [169,170,171,172,173,174,175,176,177,178,179,180,181,182,183,184,185,186,187,188,189,190,191,192,193,194,195] are cited in the supplementary file.
