# Supplementary material for: Predicting Alcohol-Related Memory Problems in Older Adults: A Machine Learning Study with Multi-Domain Features
Source: Behav Sci (Basel). 2023 May 18;13(5):427. doi: 10.3390/bs13050427 (PMC10215235; doi:10.3390/bs13050427)
Supplement: Supplementary file 1 [file behavsci-13-00427-s001.zip › behavsci-2308842-supplementary.pdf]

Supplementary Material: "Predicting Alcohol-Related Memory Problems in Older Adults: A Machine Learning Study with Multi-Domain Features"

S1. Material and Methods

S1.1. Sample Description

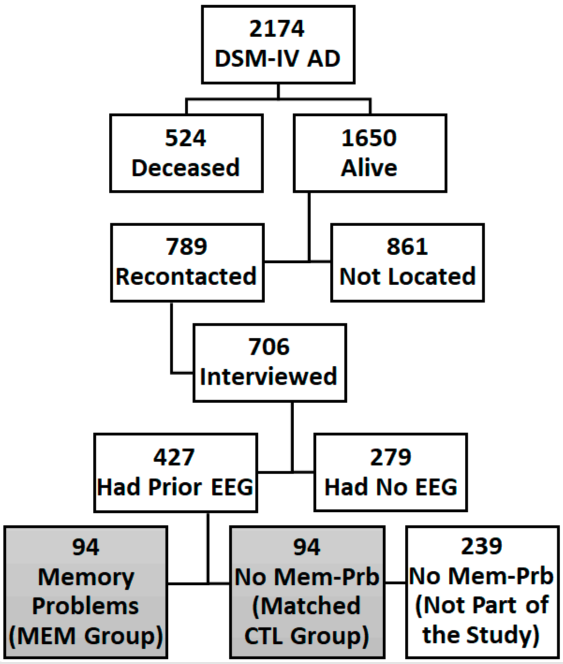

Figure S1. Flowchart showing the selection process for the study sample. ***Abbreviation:*** **AD – Alcohol dependence; MEM – Memory Group; CTL – Comparison Group; Mem-Prb – Memory Problems.**

The data were collected from six collection centers of the COGA study (SUNY Brooklyn, University of Connecticut, Indiana University, Washington University in St. Louis, University of Iowa, and University of California, San Diego). A phone interview was conducted to assess current functional and health status, including alcohol use and memory problems. All living participants provided informed consent in compliance with their local IRBs. The flow chart for the sample selection for the current study is illustrated in **Figure S1**. Initially, we identified 2174 older COGA participants who: (i) met the criteria for lifetime DSM-IV alcohol dependence based on the Semi-Structured Assessment for the Genetics of Alcohol (SSAGA) [49,50] based on prior interviews during earlier phases of COGA, (ii) were born before 1967 (thus to be aged 50 or older at the assessment), and (iii) had DNA collected. Circumstances dictated that the study be conducted over a 12-month period starting in January 2017. Out of the 2174 eligible participants, 524 were deceased, and of those that were alive, 789 were contacted (861 were not contacted for various reasons). Out of the 789 who were re-contacted, 706 were administered a brief telephone interview for a recent follow-up study. The present study sample was selected from 427 individuals who had prior EEG recordings during the earlier phases of COGA more than 18 years ago (Mean=18.53; SD=3.86). Among those with EEG, 94 individuals, who had endorsed having alcohol-related memory problems during the past 5 years and/or 10 years (self-report) during the recent follow-up telephone interview, formed the experimental group with memory problems (*Memory group*). The matched comparison control group (*Control group*, N=94) was selected from those with EEG but without having any reported memory problems by matching based on several parameters: age at EEG recording, sex, ethnicity, and past alcohol use pattern during the latest SSAGA interview.

S1.2. Recent Follow-up Telephone Interview

The items of the recent follow-up telephone interview covered the following categories: (a) marital status, (b) living arrangements, (c) education, (d) employment, (e) physical health, (f) mental health; (g) alcohol use and related health consequences (quantity/frequency, memory, blackouts, etc.); and (h) willingness to participate again in a future assessment. Alcohol-related questions consisted of prior 5-year and 10-year alcohol problems that included alcohol-related blackouts, difficulties maintaining drinking limits, spending much significant time using or recovering from its effects, interpersonal or work/school problems, use in hazardous situations, problems cutting back or stopping use, alcohol-related health impairment, and signs of alcohol withdrawal. Items about the quantity/frequency of alcohol use were queried for the past 12 months before the interview. Self-ratings of current physical and mental health were each scored on a 4-point scale from excellent to poor, and self-rating of memory on a 3-point scale of better, the same, or worse compared to other people their age. Additional details of the interview items and related data are available from our previous publications [44,45]. The list of variables from the follow-up interview schedule (N=12) is listed below in **Table S1**.

Table S1: The list of variables from the follow-up interview schedule (N=12) included in the random forest classification model.

Feature	Description	Instrument / Source
PhyHealth	The current state of physical health	Followup interview schedule
MenHealth	The current state of mental health	Followup interview schedule
DaysLastDrk	Days since the last full standard drink	Followup interview schedule
WeeksDrk	in the past 12 months, the number of weeks with alcohol consumption (max=52 weeks)	Followup interview schedule
Drk24Hr	In the past 12 months, the largest number of drinks in 24 hours period	Followup interview schedule
DaysAbst	In the last 12 months, the longest period without drinking (in number of days)	Followup interview schedule
AlcExp5yrs	Number of alcohol-related negative experiences or symptoms in the last 5 years (max=6)	Followup interview schedule
AlcWthSx5yrs	Number of alcohol withdrawals symptoms in the last 5 years (max=5)	Followup interview schedule
AlcHlthProb5yrs	Number of health problems in the last 5 years (max=5)	Followup interview schedule
AlcExp10yrs	Number of alcohol-related negative experiences or symptoms in the last 10 years (max=6)	Followup interview schedule
AlcWthSx10yrs	Number of alcohol withdrawals symptoms in the last 10 years (max=5)	Followup interview schedule
AlcHlthProb10yrs	Number of health problems in the last10 years (max=5)	Followup interview schedule

S1.3. EEG Data Acquisition and Preprocessing

Prior to the EEG recording, participants were asked to have abstained from alcohol for a minimum of 5 days. Individuals were excluded from the recording if they reported any of the following: (1) recent alcohol use in the past 5 days (i.e., positive breath-analyzer test); (2) hepatic encephalopathy/cirrhosis of the liver; (3) significant history of head injury, seizures or neurosurgery; (4) uncorrected sensory deficits; (5) taking medication known to influence brain functioning; and (6) other acute/chronic medical/neurological illnesses that affect brain function (multiple sclerosis, meningitis, encephalitis, neurodegenerative diseases, stroke, traumatic brain injury, brain tumor, etc.). Participants were seated comfortably in a dimly lit sound-attenuated, temperature-regulated booth (Industrial Acoustics, Bronx, NY, USA). EEG was recorded during the awake, eyes-closed resting state for 4.25 minutes, either using a MASSCOMP 5550 system (Concurrent Computer Corporation, Duluth, GA, USA) at the sampling rate of 256 Hz with bandpass between 0.02–50 Hz or using a Neuroscan system (Version 4.1) (Compumedics Limited, Charlotte, NC, USA) at a sampling rate of either 500 Hz or 512 Hz with bandpass between 0.02–100.0 Hz. EEG signals were recorded using an electrode cap (Electro-Cap International, Eaton, OH, USA) with a 19-channel montage of the 10–20 international system [169-171], and were amplified 10,000 times by either Sensorium (Charlotte, VT, USA) EPA-2 or Neuroscan amplifiers. The reference electrode was fixed on the nose tip, and a forehead electrode served as the ground. The electrooculogram (EOG) was recorded by a supraorbital vertical electrode and by a horizontal electrode on the external canthus of the left eye. Electrode impedances were maintained below 5 kΩ. EEG acquisition protocol was identical across all six collection sites of COGA [46,172].

As described in our previous work on EEG source functional connectivity [58], preprocessing was performed using custom scripts in Matlab (The MathWorks, Inc., Natick, MA) at two levels: (a) on the entire continuous EEG recording and (b) on each of the segmented epochs. The following steps were performed on the entire continuous EEG trace of the recording: (i) data points were resampled to 256 Hz for harmonizing different sampling rates; (ii) bandpass filtering at 0.05–50 Hz to keep only the frequency range of interest; (iii) waveforms were "detrended" to remove low-frequency components resulting in a near linear upward/downward trending deviation; and (iv) "de-meaning" was done by subtracting the gross mean of the entire EEG trace from each data point in order to align the waveforms close to the zero-amplitude baseline. Then, the continuous EEG data were segmented into epochs of 2000 ms. The next batch of preprocessing steps was performed on each of the epochs: (i) detrending; (ii) baseline alignment by subtracting epoch mean from each data point; (iii) interpolation of missing data or "flat" channels by computing the mean of surrounding nearest channels; (iv) removal of epochs with DC shift/drift involving voltage steps higher than 75 mV between any two adjacent sampling points; and (v) removal of possible EOG contaminated epochs if any data point was beyond the threshold of ±100 µV or if the difference between lowest and highest amplitude within the epoch was 200 µV. Artifact free 30 artifact-free random epochs were selected randomly for the functional connectivity analysis to keep a uniform minimum number of epochs across subjects.

S1.4. EEG Functional Connectivity Analysis using eLORETA

The eLORETA software [22,55] includes several different methods to analyze EEG source data (current density) in 3-dimensional space with 6239 voxels at 5×5×5 mm spatial resolution. The functional connectivity across the default mode network regions (**Figure S2**) was computed using the EEG source data based on lagged linear connectivity (LLC), which is less susceptible to volume conduction artifacts of the scalp-recorded EEG signals (Pascual-Marqui et al, 2007). The procedure adopted in the study has been detailed in our previous publications [58,59]. Lagged phase synchronization is a measure of similarity (a corrected phase synchrony value) between signals in the frequency domain based on normalized Fourier transforms [22], representing the strength of connectivity between two signals by subtracting the instantaneous zero-lag (non-physiological) contribution from the total connectivity to retain only the physiological connectivity between true cortical sources [22,23]. As explained by Canuet et al. [23], the classical coherence or connectivity measure, which contains both real and imaginary components, is represented as:

(i) $$\varphi^2_{x,y}(\omega) = \left|f_{x,y}(\omega)\right|^2 = \left\{\mathrm{Re}\left[f_{x,y}(\omega)\right]\right\}^2 + \left\{\mathrm{Im}\left[f_{x,y}(\omega)\right]\right\}^2$$, in which

(ii) $$f_{x,y}(\omega) = \frac{1}{N_R}\sum_{k=1}^{N_R}\left[\frac{x_k(\omega)}{|x_k(\omega)|}\right]\left[\frac{y^*_k(\omega)}{|y_k(\omega)|}\right]$$

On the other hand, the Lagged phase synchronization, which statistically partials out the instantaneous component of the total connectivity, is defined as:

$$\varphi^2_{x,y}(\omega) = \frac{\{\mathrm{Im}[f_{x,y}(\omega)]\}^2}{1-\{\mathrm{Re}[f_{x,y}(\omega)]\}^2} \quad \text{(iii)}$$

In equations (i) and (iii), *Re* and *Im* denote the real and imaginary parts of a complex number. In equation (ii), xk(ω) and yk(ω) denote the discrete Fourier transforms of x and y at frequency ω for the k-th EEG segment or epoch (k=1…NR), in which NR is the number of epochs. Thus, lagged phase synchronization, which is devoid of the instantaneous component of the classical coherence measure, is a powerful measure to elicit uncontaminated functional connectivity between the signals of interest.

S1.5. Functional Connectivity Across the Default Mode Network

The default mode network regions analyzed in the study are illustrated in **Figure S2**. Each seed region contained voxels within a 10 mm radius from the peak/centroid point of the region. The ROI-to-ROI connectivity [173], the most commonly used method to derive functional connectivity across brain regions [174], was computed using the exact Low Resolution Electric Tomography software (eLORETA) software [22,55] for the custom frequency bands: delta (1–3 Hz), theta (4–7 Hz), alpha (8–12 Hz), beta (13–29 Hz), and gamma (30–40 Hz).

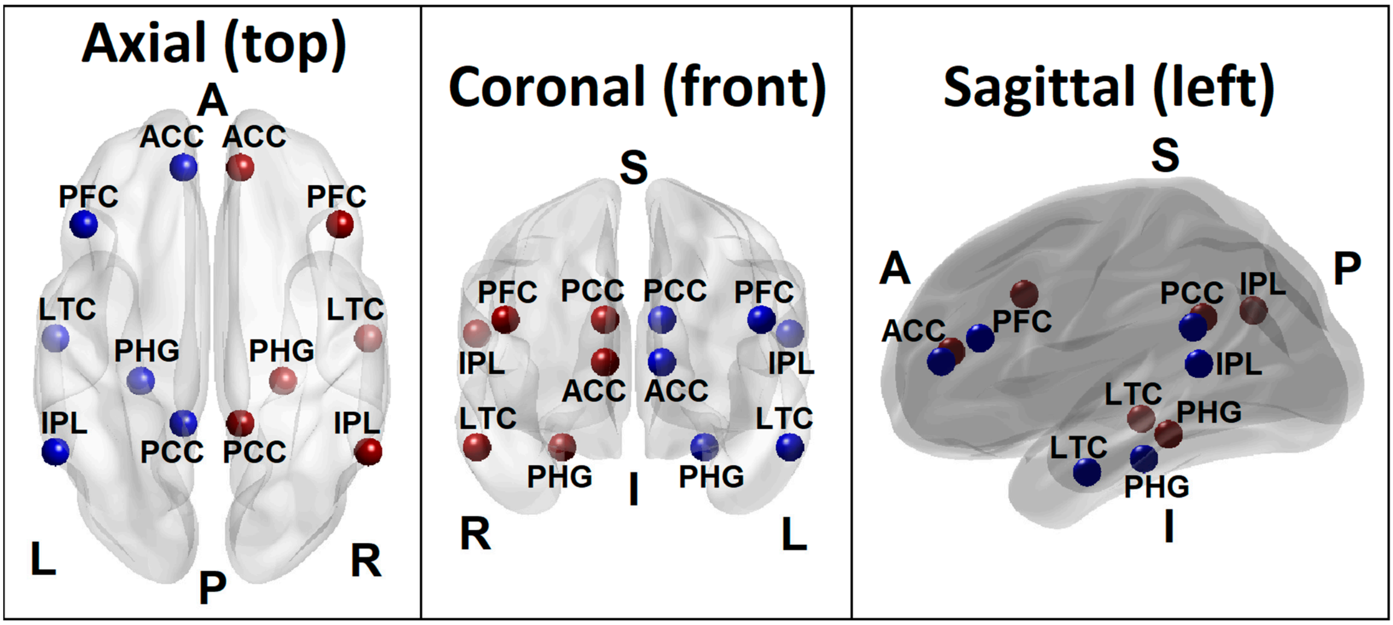

Figure S2. The Default Mode Network nodes included for the computation of functional connectivity: Six bilateral default mode network nodes from which functional connectivity was calculated include: prefrontal cortex (PFC), anterior cingulate cortex (ACC), posterior cingulate cortex (PCC), hippocampal formation (HCF), inferior parietal lobule (IPL), and lateral temporal cortex (LTC) in each hemisphere. [Views: Top view–left panel; Front view–middle panel; Side view–right panel. Direction: A–Anterior; P–Posterior; L–Left; R–Right; S–Superior; I–Inferior]

S1.6. Assessment of Temperament, Personality, and Alcohol Experience

The seven questionnaires from which behavioral data included for the current study were: (i) Sensation Seeking Scale (SSS) Form-V [175], a 40-item self-report measure consisting of four subscales that include thrill and adventure seeking (TAS), experience seeking (ES), disinhibition (Dis), and boredom susceptibility (BS); (ii) Tri-Dimensional Personality Questionnaire (TPQ) [176], a 100-item self-report measure to assess novelty seeking (NS), harm avoidance (HA), and reward dependence (RD); (iii) Daily Hassles and Uplifts (DHU) [177], a 53-item scale to measure cumulative indices of hassles (HSL) and uplifts (UPL); (iv) NEO Five Factor Inventory (NEO) [178], a 60-item self-report questionnaire that measures five domains of adult personality, including such as neuroticism (N), extroversion (E), openness to experience (O), agreeableness (A), and conscientiousness (C); (v) Perceived Social Support (PSS) [179] to measure perceived support from family (PSS-FA) and friends (PSS-FR); (vi) Alcohol Expectancy Questionnaire Adult (AEQ) [180], a 120-item self-report form to measure the respondent's beliefs about the effects of alcohol on global positive changes (GPC), enhanced sexuality (ES), physical and social pleasure (PSP), increased social assertiveness (ISA), arousal and aggression (AA), and relaxation and tension reduction (RTR); and (vii) Self Rating of Response to Ethanol (SRE) [181], a self-report instrument to measure subjective and actual effects of drinking for the first 5 drinking episodes (SRE-5drk), first 3 months of regular drinking (SRE-3mon), and period of heaviest drinking (SRE-Hvy). These variables are listed in **Table S2** below.

Table S2: The list of variables (N=27) for the personality and life experiences questionnaires.

pala	Instrument	Details
**SSV_DIS**	Sensation Seeking Scale Form-V (SSV)	SSV score for Disinhibition (DIS) subscale
**SSV_BS**	Sensation Seeking Scale Form-V (SSV)	SSV score for Boredom Susceptibility (BS) subscale
**SSV_TAS**	Sensation Seeking Scale Form-V (SSV)	SSV score for Thrill and Adventure Seeking (TAS) subscale
**SSV_ES**	Sensation Seeking Scale Form-V (SSV)	SSV score for Experience Seeking (ES) subscale
**SSV_TOT**	Sensation Seeking Scale Form-V (SSV)	SSV Total score
**TPQ_NS**	Tri-Dimensional Personality Questionnaire (TPQ)	TPQ score for Novelty Seeking (NS) category
**TPQ_HA**	Tri-Dimensional Personality Questionnaire (TPQ)	TPQ score for Harm Avoidance (HA) category
**TPQ_RD**	Tri-Dimensional Personality Questionnaire (TPQ)	TPQ score for Reward Dependence (RD) category
**AEQ_GPC**	Alcohol Expectancy Questionnaire Adult (AEQ)	AEQ score for Global Positive Changes (GPC) subscale
**AEQ_ES**	Alcohol Expectancy Questionnaire Adult (AEQ)	AEQ score for Enhanced Sexuality (ES) subscale
**AEQ_PSP**	Alcohol Expectancy Questionnaire Adult (AEQ)	AEQ score for Physical and Social Pleasure (PSP) subscale
**AEQ_ISA**	Alcohol Expectancy Questionnaire Adult (AEQ)	AEQ score for Increased Social Assertiveness (ISA) subscale
**AEQ_RTR**	Alcohol Expectancy Questionnaire Adult (AEQ)	AEQ score for Relaxation and Tension Reduction (RTR) subscale
**AEQ_AP**	Alcohol Expectancy Questionnaire Adult (AEQ)	AEQ score for Arousal and Aggression (AA) subscale
**DHU_HSL**	Daily Hassles and Uplifts (DHU)	DHU score for Hassles (HSL) category
**DHU_UPL**	Daily Hassles and Uplifts (DHU)	DHU score for Uplifts (UPL) category
**NEO_N**	NEO Five Factor Inventory (NEO)	NEO score for Neuroticism (N) category
**NEO_E**	NEO Five Factor Inventory (NEO)	NEO score for Extroversion (E) category

**NEO_O**	NEO Five Factor Inventory (NEO)	NEO score for Openness to experience (O) category
**NEO_A**	NEO Five Factor Inventory (NEO)	NEO score for Agreeableness (A) category
**NEO_C**	NEO Five Factor Inventory (NEO)	NEO score for Conscientiousness (C) category
**SRE_5drk**	Self Rating of Response to Ethanol (SRE)	SRE score for the effects of drinking during the first 5 drinking occasions (5drk)
**SRE_Hvy**	Self Rating of Response to Ethanol (SRE)	SRE score for the effects of drinking during the heviest drinking period (Hvy)
**SRE_3mon**	Self Rating of Response to Ethanol (SRE)	SRE score for the effects of drinking during the last 3 months (3mon)
**SRE_Tot**	Self Rating of Response to Ethanol (SRE)	SRE total score
**PSS_Fam**	Perceived Social Support (PSS)	PSS score for the Family (Fam) scale
**PSS_Frs**	Perceived Social Support (PSS)	PSS score for the Friends (Frs) scale

S1.7. Genomic Data and Polygenic Risk Scores (PRS)

Genotyping of the COGA data was conducted across different phases of data collection, and genotyped at multiple sites, including (i) Center for Inherited Disease Research using the Illumina HumanHap1M array [182]; (ii) Genome Technology Access Center at Washington University School of Medicine using the Illumina OmniExpress [183]; and (iii) Rutgers University using the Affymetrix Smokescreen array [184]. Data were imputed to 1000 Genomes (Phase 3, version 5) using SHAPEIT [185] and then Minimac3 [186]. Genotyping arrays were imputed separately due to different variant contents on each array. Prior to imputation, variants with missing rates > 5%, MAF < 3%, and HWE p values <0.0001 were excluded. Following imputation, genotype probabilities ≥ 0.90 were changed to genotypes. Mendelian errors in the imputed SNPs were reviewed and resolved as described previously [187,188]. SNPs with an imputation information score < 0.30 or MAF < 0.03 were excluded from subsequent analysis.

The phenotypes for which PRS calculations were performed were the following: (i) AUD diagnosis based on ICD-9 or ICD-10 codes [60], (ii) AUDIT-C scores [60], and (iii) maximum habitual alcohol intake [61] from the Million Veteran Program (MVP), and DSM-IV alcohol dependence [62] from the Psychiatric Genomic Consortium (PGC). Each of these GWAS had samples from both European Ancestry (EA) and African Ancestry (AA). However, PRS of neurocognitive phenotypes, specifically memory functions, was not included in the study due to a lack of availability of multi-ethnic PRS data. The PRS-CSx [63,64,66,67], used in the current study, is an extension of PRS-CS [63] and has been primarily implemented for cross-ethnic polygenic prediction. This method integrates summary statistics of GWAS and external LD reference panels from multiple populations to improve cross-population polygenic prediction. For the current study, we used only the SNPs that were common to both European and African ancestries. We also limited the SNPs for score creation to HapMap3 SNPs that overlapped between the original GWAS summary statistics, the LD reference panels (1000 Genomes Phase III European and African subsamples), and the target samples for score creation. PRS were converted to Z-scores for interpretability.

S1.8. Feature selection of EEG Functional Connectivity variables

In the current study, the feature selection method was used as a first stage to reduce irrelevant and redundant variables which may otherwise add noise to the predictive models [70-72]. Advantages of feature selection include a better understanding of the data quality, minimal computation requirements, reducing the effect of the curse of dimensionality (problems, such as sparsity, related to high-dimensional datasets), and also improving predictor performance [71]. In the current study, we applied binomial lasso regression as the feature selection method as implemented in R-package 'glmnet' [189], in which generalized linear (used here) and similar models are fitted via penalized maximum likelihood, and the regularization path is computed for the lasso (used here) or elastic net penalty at a grid of values for the regularization parameter *lambda*. In the first step, a binomial logistic regression model of classification type involving model fit criteria

(e.g., 10% yield). In the second step, beta coefficients for a specific lambda criterion ("lambda.min" or "lambda.1se") are derived and only the features with "non-zero" coefficients (i.e., the features with signals or classificatory power) are selected. Using this method, a set of EEG functional connectivity variables with significant predictive values to discriminate the *Memory* group from the *Control* group. The method adopted in the current analysis is based on the Lasso method as implemented in Fonti and Belitser [190], in which the model included the maximum output features "pmax" was set to 10% (i.e., 330 ÷ 10% = 33 variables). The 10-fold cross-validation, coupled with lambda thresholding at 1 SE (λ_{1se}), was used to extract the final set of key variables, while the area under the curve (AUC) was used to assess the classification performance of selected features.

S1.9. Random Forests classification model and parameters

Random Forests, an efficient predictive algorithm, was devised by Breiman [191]. The classifier algorithm consists of a collection of tree-structured classifiers where each tree casts a unit vote for a class/group for each set of predictor variables. A growing number of studies in computational biology are using Random Forests because of several advantages of the method. According to Qi [192], the Random Forests method is not only nonparametric but is interpretable and efficient. Further, the Random Forests method can be applied to data with small sample sizes, multi-dimensional variables, and multiple layers/levels without compromising its prediction accuracy [192]. In a large-scale benchmark experiment, the Random Forests algorithm was found to perform better than logistic regression in terms of prediction accuracy [193]. The two main parameters of the Random Forests algorithm are the number of trees in the ensemble and the number of variables randomly selected for the splitting decision at each node. Two levels of randomness are used by the Random Forests to construct the ensemble of trees: first, the model trains itself using training data for creating each tree based on bootstrap aggregating (bagging). At the second level, the algorithm randomly selects a subset of features to split at each node while growing a decision tree for group classification. To maximize the classification accuracy (by reducing the errors or impurity), only a single best feature (variable) among a random subset of features is selected at each internal node. This process is recursively repeated until one of the three conditions is met: (i) the tree has either reached a specified depth (i.e., number of layers of splits between the original data and the data at the bottom of the decision tree), (ii) the number of samples in a node becomes lower than the set threshold, and (iii) when all the samples are grouped into the same category [194]. Some of the important concepts and parameters of the Random Forests classification method are listed in *Box S1*.

Box S1: Concepts and parameters used in the Random Forest classification method

- **Trees:** Decision trees whose results are aggregated into one final result for classifying the factors or outcomes. Each tree is constructed based on a random (bootstrapped) subsample of the observations.
- **Node:** A point in a tree, where a split occurs as a result of a 'test' on an attribute leading to binary outcomes (e.g., whether a coin flip results in head or tail). A binary split at a node partitions the data from the parent node into two daughter nodes.
- **Branch:** The outcome of the test resulting in a split or two branches in a classification tree.
- **Leaf:** A terminal node that has no children or branches.
- **Random Forest Ensemble:** Aggregation of individual decision trees in order to combine predictions (votes) from each tree. The class/group/outcome with the most votes becomes the Random Forests model's prediction.
- **Bagging:** It's the short form of 'bootstrap aggregating', which is a method to improve classification by combining classifications of randomly generated training sets.
- **Out-of-bag (OOB) estimate:** The observations that are not part of the bootstrap subsample are referred to as out-of-bag (OOB) observations. The OOB error refers to the classification error based on

this subsample and serves as a validation of Random Forest model accuracy.

- **Gini (mean) decrease:** It represents the importance of a specific feature/predictor/variable (Vi) for the classification or prediction. It's the mean decrease in node impurity (classification error) of Vi. A higher Gini decrease indicates higher variable importance for Vi.
- **Accuracy decrease:** Mean decrease in prediction accuracy after Vi is not taken into account.
- **Mean minimal depth:** It refers to the number of nodes along the shortest path from the root node down to the nearest leaf node. A smaller depth for the Vi indicates its higher importance.
- **Mtry:** A preset number of features/variables/predictors randomly selected (from the entire list) for splitting at each node in the construction of each decision tree.
- **ntree:** A preset total number of trees to grow for a given model. Larger 'ntree' normally produces more stable models and more reliable predictions.
- **Number of nodes:** Total number of nodes that use Vi for splitting (it is usually equal to the number of trees if trees are shallow).
- **Times a root:** Total number of trees in which Vi is used for splitting the root node (i.e., the whole sample is divided into two based on the value of Vi).
- **P-value:** Probability value of hypothesis testing based on a one-sided binomial test that indicates whether the observed number of successes (number of nodes in which Vi was used for splitting) exceeds the theoretical number of successes if they were random.

To compute prediction error and classification accuracy, we used the Out-of-Bag (OOB) error estimate, which represents the classification error obtained from the out-of-bag sample (about one-third of the total sample) that was not part of the bootstrap sample (about two-thirds of the total sample) used in growing the forests. In the Random Forests model, cross-validation in a separate test sample is not required, as it is estimated internally in the algorithm [195]. During each iteration of constructing a decision tree, about two-thirds of the bootstrap sample from the training data is used, and about one-third of the sample is left out during each bootstrap process, which is called the out-of-bag (OOB) sample. The classification error calculated from this sample is called the OOB error score. The aggregate of OOB scores from all decision trees will provide the ensemble OOB error rate (i.e., classification error) as well as the Random Forests model accuracy rate for the Random Forests model. Thus, the OOB score provides validation for the Random Forests model. The Random Forests classification model included 29 default mode network connections from feature selection (see Results section), 27 variables on temperament, personality, and life experiences, 12 variables on health and alcohol-related problems, and 4 PRS scores on alcohol phenotypes as features, while the group status (*Memory* vs. *Control* group) served as the outcome variable. In the model, the maximum number of trees 'ntree' was set at 500. The optimal number of features analyzed at each node ('Mtry') was estimated to be 9 (using the 'tuneRF' function) and was used in the classifier algorithm. The final list of variables that significantly contributed to the classification was tabulated, and 3-dimensional connectivity maps of top significant default mode network connections within a brain anatomical template were created using custom Matlab scripts.

S2. Results

S2.1. Feature Selection of EEG Functional Connectivity Variables

The feature selection procedure identified a total of 29 functional connectivity variables from multiple frequency bands connecting across the twelve default mode network seeds (see **Table S3** below**)**. These connections are: Delta – 12 connections (1–5, 1–6, 1–12, 2–12, 2–5, 3–5, 3–7, 4–8, 5–6, 6–11, 7–11, 8–12), Theta – 6 connections (2–5, 2–11, 4–10, 4–6, 6–10, 9–11), Alpha – 4 connections (2–5, 2–11, 7–10, 7–12), Beta – 5 (1–4, 2–10, 3–7, 4–9, 5–12), and Gamma – 2 variables (2–10, 4–12).

Table S3: The list of functional connectivity variables (N=29) that were identified by the feature selection method and included in the random forest classification analysis.

Feature	Frequency	Node 1	Node 2
FC_De_1_5	Delta	Left posterior cingulate cortex	Left inferior parietal lobule
FC_De_1_6	Delta	Left posterior cingulate cortex	Right inferior parietal lobule
FC_De_1_12	Delta	Left posterior cingulate cortex	Right parahippocampal gyrus
FC_De_2_5	Delta	Right posterior cingulate cortex	Left inferior parietal lobule
FC_De_2_12	Delta	Right posterior cingulate cortex	Right parahippocampal gyrus
FC_De_3_5	Delta	Left anterior cingulate cortex	Left inferior parietal lobule
FC_De_3_7	Delta	Left anterior cingulate cortex	Left prefrontal cortex
FC_De_4_8	Delta	Right anterior cingulate cortex	Right prefrontal cortex
FC_De_5_6	Delta	Left inferior parietal lobule	Right inferior parietal lobule
FC_De_6_11	Delta	Right inferior parietal lobule	Left parahippocampal gyrus
FC_De_7_11	Delta	Left prefrontal cortex	Left parahippocampal gyrus
FC_De_8_12	Delta	Right prefrontal cortex	Right parahippocampal gyrus
FC_Th_2_5	Theta	Right posterior cingulate cortex	Left inferior parietal lobule
FC_Th_2_11	Theta	Right posterior cingulate cortex	Left parahippocampal gyrus
FC_Th_4_6	Theta	Right anterior cingulate cortex	Right inferior parietal lobule
FC_Th_4_10	Theta	Right anterior cingulate cortex	Right lateral temporal cortex
FC_Th_6_10	Theta	Right inferior parietal lobule	Right lateral temporal cortex
FC_Th_9_11	Theta	Left lateral temporal cortex	Left parahippocampal gyrus
FC_Al_2_5	Alpha	Right posterior cingulate cortex	Left inferior parietal lobule
FC_Al_2_11	Alpha	Right posterior cingulate cortex	Left parahippocampal gyrus
FC_Al_7_10	Alpha	Left prefrontal cortex	Right lateral temporal cortex
FC_Al_7_12	Alpha	Left prefrontal cortex	Right parahippocampal gyrus
FC_Be_1_4	Beta	Left posterior cingulate cortex	Right anterior cingulate cortex
FC_Be_2_10	Beta	Right posterior cingulate cortex	Right lateral temporal cortex
FC_Be_3_7	Beta	Left anterior cingulate cortex	Left prefrontal cortex
FC_Be_4_9	Beta	Right anterior cingulate cortex	Left lateral temporal cortex
FC_Be_5_12	Beta	Left inferior parietal lobule	Right parahippocampal gyrus
FC_Ga_2_10	Gamma	Right posterior cingulate cortex	Right lateral temporal cortex
FC_Ga_4_12	Gamma	Right anterior cingulate cortex	Right parahippocampal gyrus

Abbreviations: FC–Functional Connectivity; De–Delta; Th–Theta; Al–Alpha; Be–Beta; Ga–Gamma; Numbers in functional connectivity variables: 1-12 of the default mode network.

S2.2. Random Forests Classification Accuracy

The overall prediction accuracy of the Random Forests model to classify *Memory* and *Control* individuals using FC, PRS, behavioral, and clinical predictors, as estimated by the area under the ROC curve, was 88.29% (**Figure S3**). The confusion matrix of the Random Forests model showed that the accuracy rate for the *Memory* and *Control* groups were 72.34% and 90.43%, respectively, with an accuracy rate of 81.39%, sensitivity of 88.31%, specificity of 76.58%, positive predictive value of 72.34% and negative predictive value of 90.43%. In other words, 68 individuals in the *Memory* group and 85 individuals in the *Control* group (out of 94 in each group) were correctly identified by the Random Forests classification algorithm.

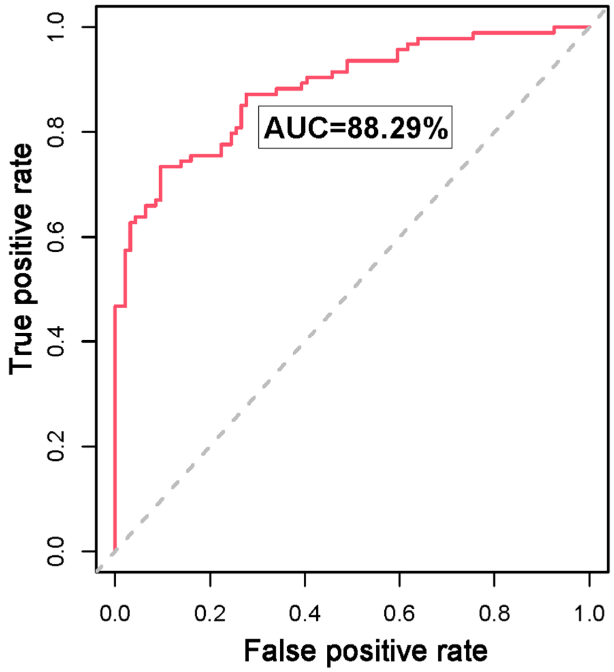

Figure S3. ROC curve (red line) derived from the Random Forests model to classify *Memory* and *Control* individuals using functional connectivity, PRS, and clinical and behavioral predictors. The predictive accuracy measured by the AUC was 88.29%. The diagonal line (dashed gray line) indicates the line of "no discrimination" and splits the area into the upper and lower half (50%).

S2.3. Top Significant Features Contributed to the Classification

The 29 significant features that were identified by the Random Forests classification and ranked based on mean minimal depth against the number of decision trees are illustrated in **Figure S4**. Minimal depth for a variable in a tree refers to the depth of the node which splits on that variable measured from the root of the tree. Lower mean minimal depth represents the higher number of observations/participants categorized into a specific group based on the variable and thus contributing to better classification accuracy, and therefore smaller depth for a feature indicates its higher importance in classification.

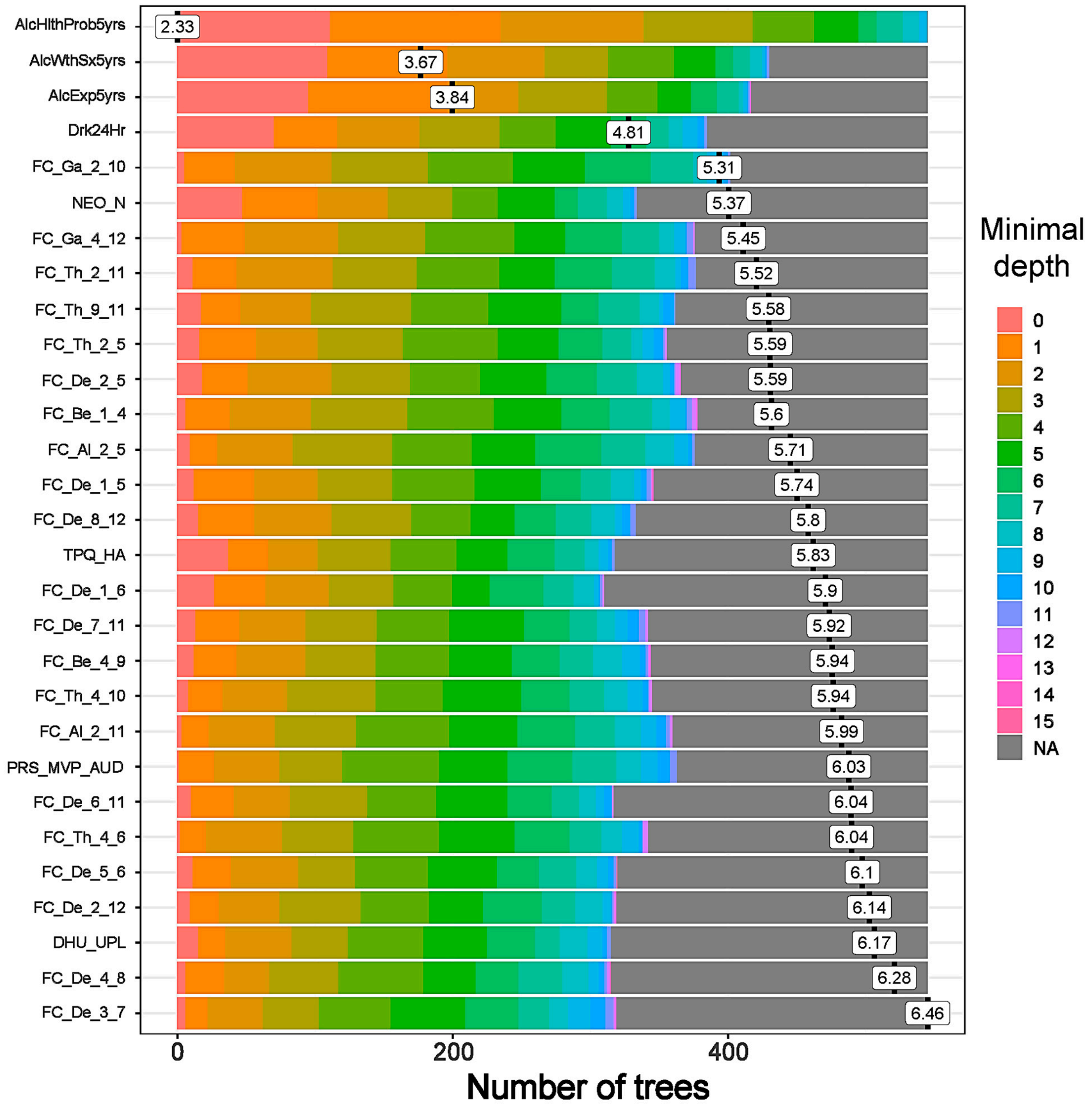

Figure S4. The distribution of minimal depth among the trees of the forests for each feature is color-coded for different levels of minimal depth. The features that contributed to classifying the *Memory* individuals from the *Control* participants are ranked in ascending order of minimal depth.

In order to determine the concordance of rankings between any two Random Forest (RF) parameters, a correlation matrix was plotted against each parameter (see **Figure S5)**. It was found that all of the Random Forests parameters of importance showed very high correlations among each other in ranking the features, suggesting a high concordance among these parameters in group classification.

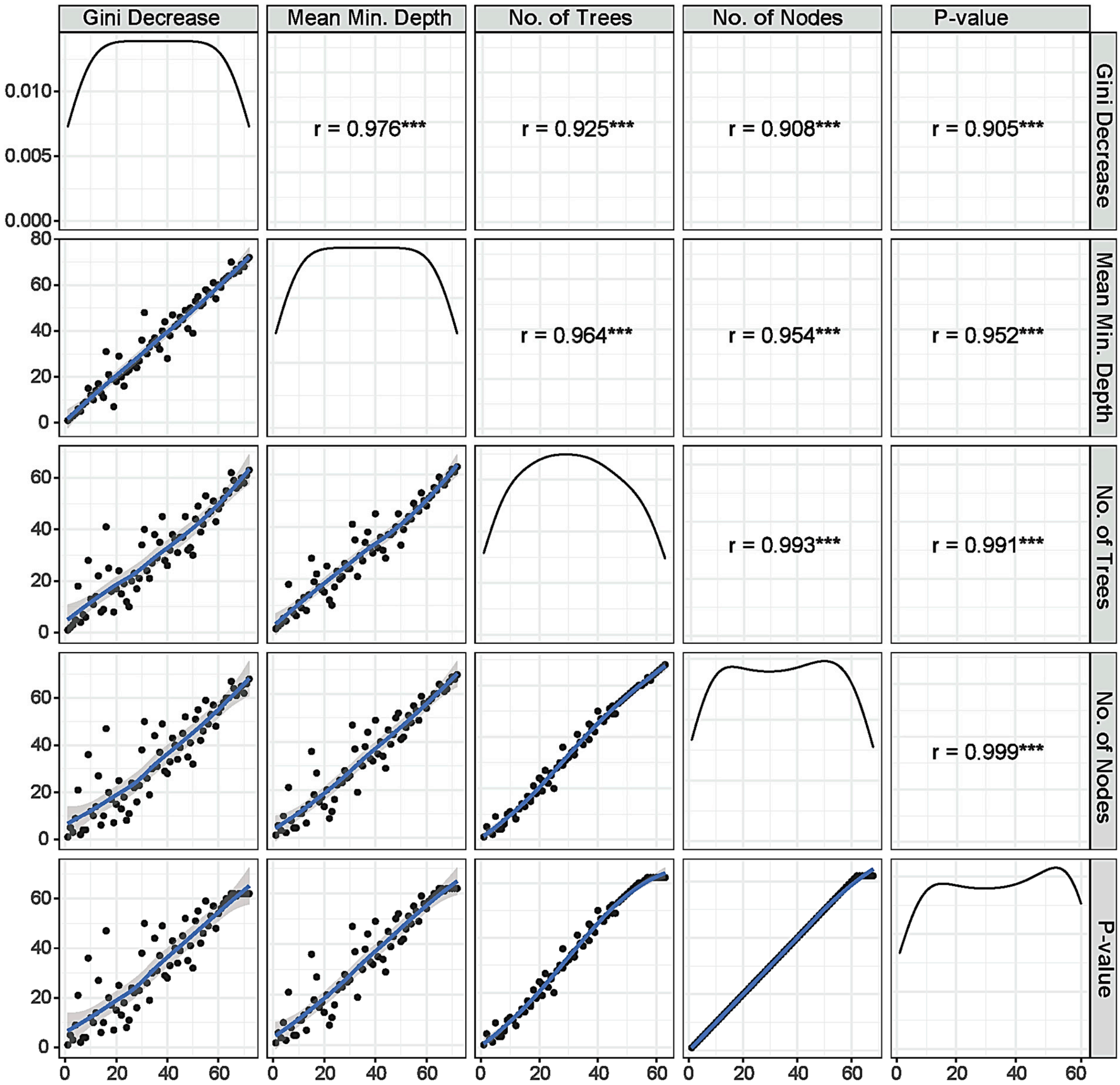

Figure S5: Concordance of rankings between any two Random Forests parameters. Panels in the lower triangle of the grid show the distribution of rankings for all 72 variables (black dots) around a trend line (blue curve). The panels in the upper triangle of the grid show a correlation coefficient between the rankings of any two parameters.
